# Enhanced cGMP Interactor Rap Guanine Exchange Factor 4 (EPAC2) Expression and Activity in Degenerating Photoreceptors: A Neuroprotective Response?

**DOI:** 10.3390/ijms23094619

**Published:** 2022-04-21

**Authors:** Michel Rasmussen, Jiaming Zhou, Frank Schwede, Per Ekström

**Affiliations:** 1 Department of Clinical Sciences Lund, Faculty of Medicine, Lund University, 22184 Lund, Sweden; jiaming.zhou@med.lu.se (J.Z.); per.ekstrom@med.lu.se (P.E.); 2 BIOLOG Life Science Institute GmbH & Co. KG, 28199 Bremen, Germany; fs@biolog.de

**Keywords:** retinal degeneration, cGMP, neuroprotective, rap guanine exchange factor 4, photoreceptors, cAMP

## Abstract

The disease retinitis pigmentosa (RP) leads to photoreceptor degeneration by a yet undefined mechanism(s). In several RP mouse models (i.e., *rd* mice), a high cyclic GMP (cGMP) level within photoreceptors is detected, suggesting that cGMP plays a role in degeneration. The rap guanine exchange factor 4 (EPAC2) is activated by cyclic AMP (cAMP) and is an accepted cGMP-interacting protein. It is unclear whether and how cGMP interacts with EPAC2 in degenerating photoreceptors; we therefore investigated EPAC2 expression and interactions with cGMP and cAMP in retinas of the *rd1* and *rd10* models for retinal degeneration. EPAC2 expression in the photoreceptor layer increased significantly during *rd1* and *rd10* degeneration, and an increase in EPAC2 interactions with cGMP but not cAMP in the *rd1* was also seen via a proximity ligation assay on histological sections. Retinal explant cultures revealed that pharmacological inhibition of the EPAC2 activity reduced the photoreceptor layer thickness in the *rd10* retina, suggesting that EPAC2 inhibition promotes degeneration. Taken together, our results support the hypothesis that high degeneration-related cGMP leads to increased EPAC2 and cGMP interactions, inhibiting EPAC2. By inference, EPAC2 could have neuroprotective capacities that may be exploited in the future.

## 1. Introduction

Retinitis pigmentosa (RP) is a disease of the eye [1,2,3] whereby the photoreceptors undergo degeneration, which causes vision impairment and potential blindness [4,5]. The prevalence of RP is 1 in 3000 to 7000 people worldwide [1,6], yet the underlying mechanism(s) behind the retinal degeneration remains unknown. General treatment options for RP are lacking, although some gene therapy studies have shown promising results and entered the clinical stage, with one current approval for Leber’s congenital amaurosis, a specific RP subtype [5,7,8]. Thus, there is a dire need to decipher the underlying mechanism(s) that give(s) rise to RP and develop more general therapies to prevent photoreceptor loss and disease progression. 

Several RP mutations [9] cause an elevation of potentially toxic cyclic GMP (cGMP) levels in the photoreceptors in a range of RP mouse models, often referred to as *rd* (retinal degeneration) mice [9,10,11,12,13]. cGMP is among the universal second messengers and has numerous interacting proteins, including cGMP-dependent protein kinase (PKG) [11,14,15,16] and cAMP-dependent protein kinase (PKA) [15], as well as CNG channels [17], phosphodiesterases, and cGMP transport proteins [16]. Among these, PKG is overactivated in connection with RP, and interventions against such overactivation can reduce photoreceptor degeneration in RP models [11,18]. Therefore, pharmacological manipulation of other cGMP-interacting proteins may result in a similar neuroprotective effect. 

In a recent study of ours, the protein rap guanine exchange factor 4 (EPAC2) was suggested to interact with cGMP [19] in the retina, including in the photoreceptors [20]. The exchange protein EPAC has two isoforms (EPAC1 and EPAC2), and whereas EPAC1 is activated by cyclic AMP (cAMP) and, to some extent, by cGMP [21,22], EPAC2 is activated by cAMP and is also an accepted cGMP-interacting protein in vitro [19]. Moreover, upon cAMP binding, EPAC induces the activation of Ras-like GTPase family members Rap1 and Rap2 [23]. This links cAMP signaling to calcium mobilization, gene transcription, kinase stimulation, and cellular functions, such as cell proliferation and cell death [24,25]. However, it remains unclear whether cGMP can bind and stimulate EPAC2 directly [21]; on the other hand, it is believed that cGMP has an inhibitory effect on EPAC2 [26]. 

In the retina, EPAC1 and EPAC2 are expressed within cell bodies in the inner nuclear layer (INL) [27]. In addition, EPAC2 is expressed in the outer nuclear layer (ONL), where it has been detected in cone cell bodies, as well as inner and outer segments (IS/OS) [27]. 

Whether EPAC1 and EPAC2 play a role in RP remains unknown, but they may, as EPAC was found to be involved in other retinal pathologies (i.e., glaucoma and macular edema) [28]. Additionally, activation of the EPAC-Rap1 pathway has been suggested to restore the blood–retinal barrier and protect against permeability problems [29]. However, the situation is complex because inhibition of EPAC1 or EPAC2 can promote cell survival of cerebral cortex neurons [30] and retinal ganglion cells [28]; however, *activation* of EPAC2 can also protect certain cells [31]. This indicates that a positive or negative role for EPAC may be dependent on the actual system, such that different neurons react differently to alterations in EPAC2 activity. Interestingly, a common aspect observed for some of the diseased cells was that their EPAC2 expression was augmented [30,31]. 

In light of the above, we investigated whether EPAC2 is, in any way, altered upon RP, whether such potential changes could be caused by cGMP interaction in the retina, and what this may mean. This was achieved using *rd* mouse models and healthy wild-type (wt) counterparts in combination with an organotypic retinal explant system.

## 2. Results

### 2.1. EPAC2 Is Expressed in Rod Inner Segments and Perikaryonal Regions

To investigate EPAC2 expression in the photoreceptors, we performed immunostainings in wt and three *rd* models (i.e., *rd1,* *rd10,* and *rd2*). However, the results from the *rd2* model did not show any significance within this study and are therefore not further mentioned in this section, although *rd2* data can be found in Figure S3.

**Figure 1:**
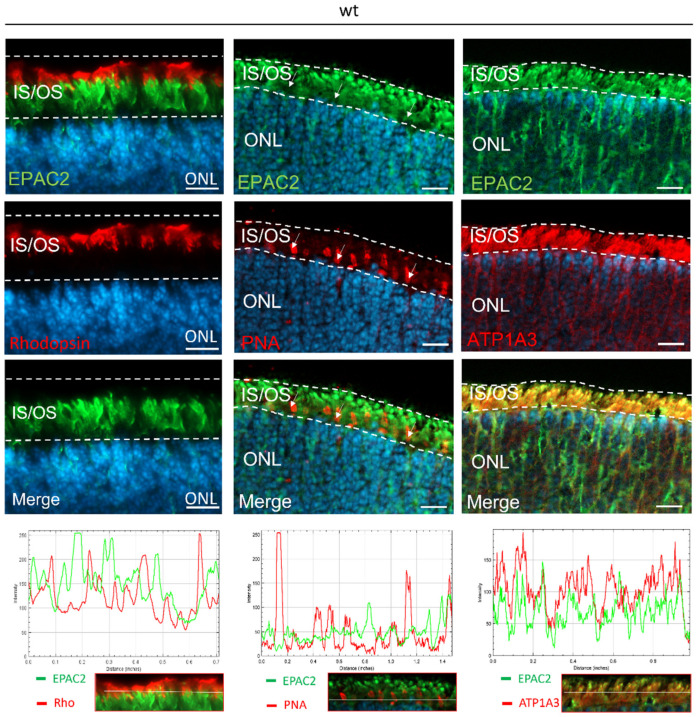
EPAC2 protein localization in wt retina. Panels show, from top to bottom, EPAC2 staining, photoreceptor markers, merged images, and fluorescence intensity profiles*. (*Left panel*) EPAC2 was expressed in the outer nuclear layer (ONL) and within photoreceptor segments (IS/OS), but there was no overlap between EPAC2 and rhodopsin, a marker for rod outer segments (OS); (*Middle panel*). There was no clear indication of colocalization between EPAC2 and peanut agglutinin (PNA, a specific glycoprotein binder that binds the extracellular matrix around the inner segments (IS) and OS, as well as the synaptic pedicles, of cones [Blanks and Johnson, 1984 [32], Johnson et al., 1986 [33]). Arrows show PNA localization; no clear colocalization is observed between PNA and EPAC2, which is further supported by the fluorescence intensity profile at the bottom. (*Right panel*) Overlap between EPAC2 and ATP1A3, a protein specific for photoreceptor IS (Molday, LL et al., 2019 [34]), as indicated by the merged picture and fluorescence intensity profiles. The pictures represent three biological replicates per staining. DAPI (blue) was used as a nuclear counterstain. Scale bar: 10 µm. * Fluorescence intensity profiles were obtained by analyzing a horizontal line (indicated by a white line in the bottom pictures) in the segments and showing the intensities of the respective green/red fluorescence in a graph.

**Figure 2:**
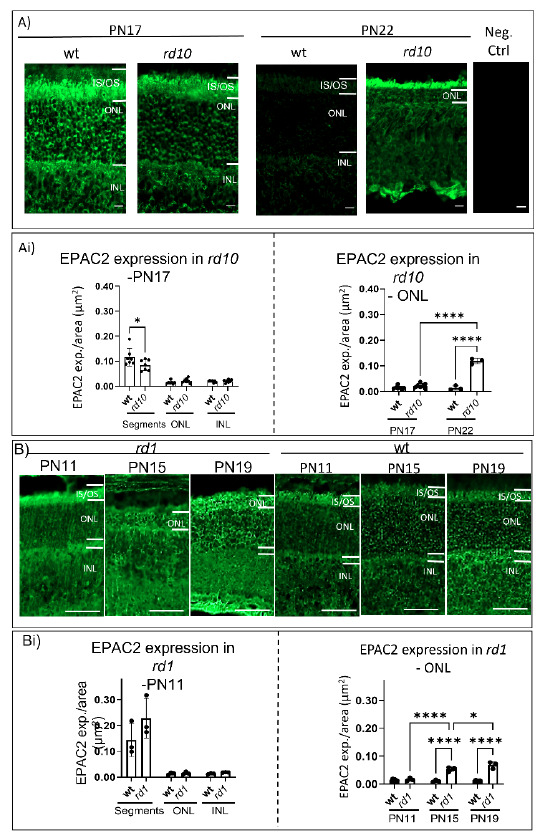
EPAC2 expression in the *rd* models compared to their respective wt retina. (**A**,**B**) Example of EPAC2 immunostaining, in this case, *rd10, rd1,* and wt retina at different ages (i.e., wt/*rd10:* PN17, and PN22, wt/*rd1:* PN11, PN15, and PN19). A negative control with no primary EPAC2 antibody is provided. Scale bar: (**A**) 10 µm and (**B**) 50 µm. Panel (**Ai**) EPAC2 expression analyses in *rd10. Left* for the different layers: segments (IS/OS), ONL, and INL; *right* data from relevant ages for *rd10* and their respective wt counterparts. (**Bi**) EPAC2 expression analyses in *rd1. Left* for the different layers: segments (IS/OS), ONL, and INL; *right* data from relevant ages for *rd1* and their respective wt counterparts. The EPAC2 expression was generally higher within the segments, and with respect to these, *rd10* had less EPAC2 expression than wt. The ONL and INL values were all significantly different from the segment values of the corresponding genotype (not labeled in the figure). Furthermore, data show EPAC2 expression increases significantly over time in both models. (**A**,**B**) Graphs represent three to seven biological replicates with mean ± SD; the two-way ANOVA was applied, and the levels of significance are * *p* < 0.05, **** *p* < 0.0001. Abbreviations the same as in Figure 1.

Staining revealed that EPAC2 was localized in the ganglion cell layer (GCL), INL, and ONL, as well as in photoreceptor segments in the mouse retina (Figure 1, Figure 2, and Figure S2). As judged from Figure 2, the staining in the ONL included a perikaryonal localization of EPAC2. A closer look reveals that the EPAC2 staining did not overlap with the rod-specific marker rhodopsin (Rho) in the segment region, although Rho seemed to be expressed in the extensions of many of the EPAC2-positive structures. EPAC2 did not colocalize with the cone-specific marker peanut agglutinin (PNA); instead, EPAC2 overlapped with the inner segment marker ATP1A3 (Figure 1). Altogether, this matches an EPAC2 expression in rod inner segments, as well as in perikaryonal regions of the photoreceptors.

### 2.2. EPAC2 Is Overexpressed upon Retinal Degeneration 

To evaluate whether EPAC2 has an altered expression during the degeneration, we performed immunostainings of retinal sections representing different ages and degeneration stages of the *rd10* and *rd1* models. The *rd* results were then compared to their respective wt counterparts (Figure 2). 

EPAC2 expression was found to be generally higher within the segments than in the ONL and INL (Figure 2Ai–Bi), and here, *rd10* expressed significantly lower EPAC2 when compared to its wt counterpart (Figure 2Bi, left). Such differences were not seen in the *rd1* model. 

We also quantified EPAC2 in the ONL over time (Figure 2Ai–Bi), including early time points (*rd10:* PN17 and *rd1:* PN11) until stages representing more advanced degeneration (*rd10:* PN22 and *rd1:* PN11 + PN19). Here, both *rd10* and *rd1* showed a significant temporal increase in EPAC2 expression within the respective model and when compared to the relevant wt (Figure 2Ai–Bi). 

### 2.3. Both cGMP and cAMP Levels Increase in Degenerating Photoreceptors 

**Figure 3:**
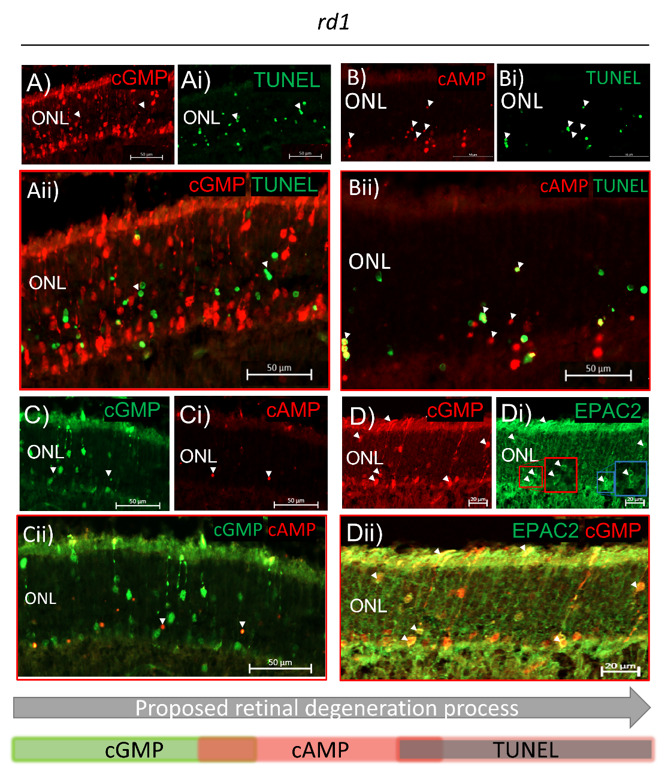
cGMP, cAMP, and EPAC2 are associated with cell death. All pictures represent the *rd1* retina at PN11. A to D all show: X, first staining; Xi, second staining; Xii, merged stainings; and increased magnification. (**A**–**Aii**) Accumulated cGMP did not overlap with TUNEL^+^ cells in the *rd1* retina. Arrowheads in A and Ai indicates that there was no obvious overlap between the cGMP and TUNEL^+^ cells. (**B**–**Bii**) Accumulated cAMP overlapped with TUNEL^+^ cells in some cases. Arrows in Bii indicate such an overlap (yellow). (**C**–**Cii**) cGMP and cAMP showed overlap in a few photoreceptor nuclei*. A to C suggest that high intracellular cGMP is followed by cAMP accumulation, which in turn is followed by TUNEL positivity (a proposed order of events is indicated in the bottom panel). (**D**–**Dii**) Accumulated cGMP colocalized with EPAC2 in degenerating cells, where EPAC2 appeared to have higher expression. Arrowheads point to colocalization of accumulated cGMP and high EPAC2 expression. The red and blue inserted squares in Di show cells containing augmented EPAC2 expression (small, red, and blue) and magnifications of these (large, red, and blue). Micrographs are representative of three biological replicates. ONL: outer nuclear layer. As judged by the position and appearance of the stained structure.

**Figure 4:**
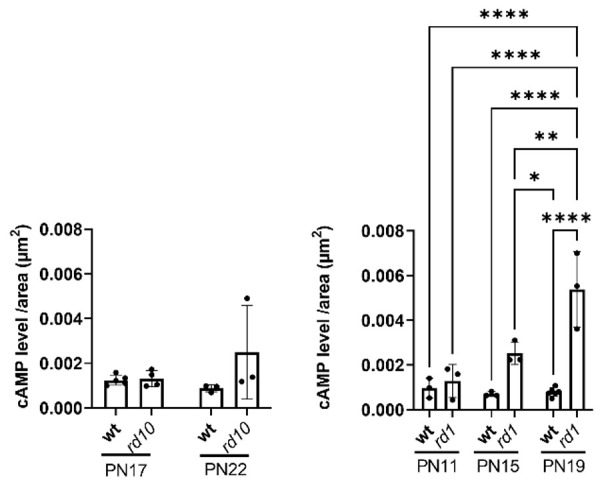
cAMP level increases within the ONL in degenerating *rd1* retinas. The cAMP level was quantified from immunofluorescence stainings from the two *rd* models and given as level/area (µm^2^): *rd10* (left) and *rd1* (right), as well as their respective wt counterparts at different ages. cAMP levels increased with time in *rd1* but were not altered significantly in *rd10* or wt. Graphs represent three to six biological replicates, and bars represent mean ± SD; levels of significance are: * *p* < 0.05, ** *p* < 0.01, **** *p* < 0.0001.

High intracellular cGMP has previously been indicated as an early degeneration marker prior to photoreceptor cell death in both *rd1* and *rd10* models [12,13]. Our results support this, as no overlap between cGMP and TUNEL-positive (TUNEL^+^) cells was detected in the ONL of the rapidly degenerating *rd1* model (Figure 3A–Aii). EPAC2-interacting cAMP has also been associated with photoreceptor degeneration [35], and our stainings showed that cAMP, like cGMP, accumulates in dying photoreceptors (Figure 3B–Bii) in a time-dependent manner (Figure 4). This is, to the best of our knowledge, the first time it has been shown that cAMP accumulates in *rd1* photoreceptors. Moreover, co-stainings with cGMP and cAMP only showed a few overlaps (Figure 3C–Cii), whereas co-staining of cAMP and TUNEL resulted in more frequent overlaps (Figure 3B–Bii), which is compatible with cAMP accumulation in the TUNEL^+^ photoreceptor nuclei. Photoreceptors that accumulated cGMP seemed, in co-stainings, to have a higher expression of EPAC2 than the non-dying cells (Figure 3D–Dii). A similar co-staining with EPAC2 and cAMP was not possible due to a clash of primary antibodies. Together, this infers that EPAC2, cGMP, and cAMP may all be relevant for the degeneration process and that their accumulation and the cell death, as indicated by TUNEL^+^, may occur in the following proposed order: cGMP → cAMP → cell death (Figure 3, bottom panel). 

### 2.4. EPAC2 Shows Increased Interaction with cGMP during Retinal Degeneration

**Figure 5:**
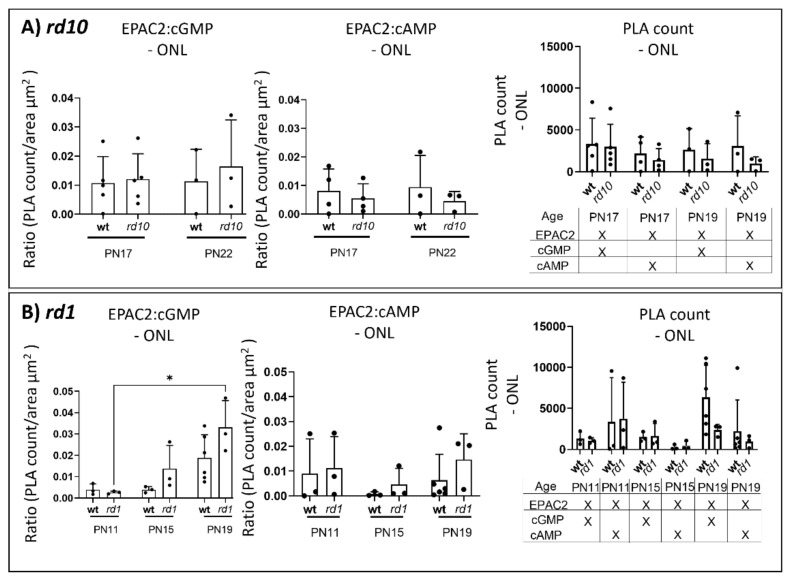
The extent of cGMP and EPAC2 proximity increases with time in the *rd* retina. Panel (**A**,**B**) gives PLA ratios (PLA counts over ONL area) and raw PLA counts for various situations: (left) proximity outcome between EPAC2 and cGMP (EPAC2:cGMP); (middle) proximity outcome between EPAC2 and cAMP (EPAC2:cAMP); (right) the raw proximity count (PLA count) was provided for comparison. (**A**) *rd10* did not show any significant alteration between EPAC2:cGMP nor between EPAC2:cAMP at PN17 or PN22. (**B**) A significant increase in the EPAC2:cGMP ratio was observed over time in the *rd1* model. Graphs represent three to six biological replicates, and bars represent mean ± SD; levels of significance are: * *p* < 0.05. ONL: outer nuclear layer.

Based on our observation that both cGMP and cAMP levels increase in the degenerating photoreceptors, we next wanted to determine whether EPAC2 would interact differently with either cGMP or cAMP during disease progression in the two different *rd* models. We therefore performed a proximity ligation assay (PLA; Figure 5, Figures S4 and S5). The quantification was calculated based on the ratio of interaction between EPAC2 and cGMP within the ONL in the *rd10* (*ratio =* number of detected EPAC2 and cGMP interactions divided by the ONL area in µm^2^; hereafter, EPAC2:cGMP; Figure 5A and Figure S4). The PLA showed no significant differences between *rd10* and wt nor over time within either of the two. However, for the *rd1* model, there was a significant ratio increase from PN11 to PN19 (Figure 5B). Interestingly, wt ONL also had, in absolute numbers, many EPAC2:cGMP interactions, particularly at PN19 (Figure 5B, right), which can be explained by the fact that wt ONL is thicker and contains more photoreceptors than *rd1* at this stage. For EPAC2 and cAMP interactions (i.e., EPAC2:cAMP, Figure 5A,B and Figure S5), the PLA revealed no significant changes between the *rd* models and their corresponding wt counterparts. 

### 2.5. Inhibiting EPAC2 Activity Reduces ONL Thickness in the rd10 Model

As previously mentioned, cGMP has an inhibitory effect [26] on EPAC2, whereas cAMP activates it [21,22]. Therefore, we wanted to determine how manipulation of the EPAC2 activity would affect the degeneration. The *rd10* model was chosen because it has a more slow-progressing degeneration, which allowed for pharmacological manipulation to be initiated before the major part of *rd10* cell death [13]; therefore, a long treatment paradigm could be applied (PN12—PN24). To evaluate possible differences in and effects on EPAC2 activity, a Rap1-GTP assay was utilized. The assay was first used on *rd10* and wt PN17 retinas, which were taken directly from animals without being cultured. Here, the *rd10* retina exhibited lower Rap1-GTP activity than the wt retina (18.85 and 38.85 arbitrary units (a.u.), respectively; Figure S6). Tests with wt explants in turn suggested that the activator S-220 and the inhibitor ESI-05 acted according to their description and caused, respectively, an increase and a decrease in Rap1-GTP compared to the untreated specimen (Figure S6). The activity of the untreated wt explants was lower than that seen in the non-explanted wt retina, suggesting that culturing as such may affect EPAC2 activity. 

**Figure 6:**
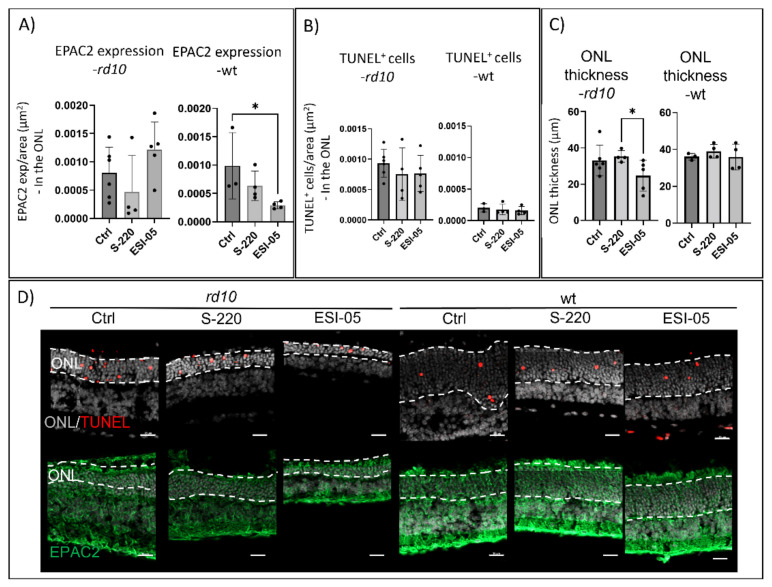
Inhibition of EPAC2 activity decreases ONL thickness in the *rd10* model. (**A**) Inhibition of EPAC2 activity by ESI-05 in *rd10* explants (treatment paradigm: PN12-PN24) caused a numerical but not significant increase in EPAC2 expression compared to the control (Ctrl, i.e., untreated retina) and the S-220-treated retina. For the wt retina, the activator S-220 reduced EPAC2 expression significantly in the ONL compared to Ctrl. (**B**) The *rd10* explants had significantly more TUNEL-positive cells compared to their healthy counterparts. No differences in the number of TUNEL^+^ cells was associated with the treatments. (**C**) ESI-05 treatment leads to a significant reduction in ONL thickness in *rd10* explants but not in wt explants. (**D**) Examples of stainings, forming the basis of the graphs above. All figures represent mean ± SD. Results represent three to six biological replicates. The Kruskal–Wallis statistical method was applied to identify statistical significance (* *p* < 0.05). The dotted line in figure C marks the ONL (outer nuclear layer). Scale bar = 20 µm.

Having established that the agents worked as expected, we next performed organotypic retinal explant culturing with retinas from wt and the *rd10* model with or without the inhibitor or the activator (Figure 6). The quantified EPAC2 expression showed that the EPAC2 inhibitor (ESI-05) significantly decreased EPAC2 expression within the ONL in the wt retina compared to the untreated control (Figure 6A). By contrast, for the *rd10* retina, the ESI-05 inhibitor produced an increased (but not statistically significant) value of EPAC2 expression when compared to the untreated control (Figure 6A). 

To reveal whether inhibition or activation of EPAC2 would influence cell death in the ONL, a TUNEL assay was performed. Here, the TUNEL^+^ cells were quantified and normalized to the ONL area. No difference in cell death was seen between *rd10* and wt with respect to activator/inhibitor conditions or no treatment (Figure 6B). To complement the TUNEL outcome, the thickness of the ONL was measured. A difference was seen in the *rd10* model, where treatment with the inhibitor resulted in an ONL thickness significantly less than that after treatment with the activator (Figure 6C), which had an ONL thickness approximately the same as the control. The wt ONL was not much thicker than that of the untreated *rd10*, which was undergoing degeneration, which can be ascribed to differences related to the use of two strains in these cases (C57BL/6J for *rd10* vs. C3H for wt). 

## 3. Discussion

### 3.1. EPAC2 Is Expressed in Rod Inner Segments and Perikaryonal Regions 

In contrast to a previous study of the normal rat retina by Whitaker et al. [27], we did not see EPAC2 localized to the outer segments in the wt mouse, nor did we observe that EPAC2 was expressed selectively within the cone cell inner and outer segments. However, we did see that EPAC2 was expressed in the inner segments of rods and in the ONL, INL, and GCL. Whitaker et al. also noted EPAC2 expression in, for instance, the INL and GCL^27^, which makes antibody differences or other technical aspects a less likely reason for the discrepant photoreceptor results and instead suggests that EPAC2 is localized differently in rat and mouse retina. 

### 3.2. EPAC2 Is Overexpressed upon Retinal Degeneration 

It was clear that although *rd1* and *rd10* have similar mutations (see below), they displayed differences in our study for some parameters. However, in concert, they support our conclusion that EPAC2 is implicated in photoreceptor degeneration. First, at an early degeneration time point, EPAC2 expression in *rd10* segments (which is limited to inner segments; see further down) was lower than in the corresponding wt segments (inner and outer), suggesting that EPAC2 may be responding to the degeneration processes. In contrast, this was not observed in *rd1,* which is likely due to inherent differences in the models, including segment status. The *rd1* retina has a fast degeneration, peaking at PN14, and has poorly developed inner and outer segments [36,37], whereas the *rd10* progression is slower, peaking around PN18. Additionally, as opposed to *rd1*, the PDE6β protein can be detected early in *rd10* segments (PN10), albeit at reduced levels compared to wt [38]. EPAC2 expression in the segments could thus be affected by genetic, as well as structural, differences during degeneration. Second, the expression of EPAC2 in the ONL, which shrinks due to cell death [12,13], was increased with time in both *rd1* and *rd10*. Regardless of the differences in EPAC2 expression in the segments, the increased EPAC2 expression in the ONL therefore connects it with degeneration. Opposite to *rd1* and *rd10*, the *rd2* model did not show any significant results in the above-mentioned experiments. However, this could be due to the models’ different mutations, where *rd1* and *rd10* have mutations in the PDE6β, a phototransduction enzyme, whereas *rd2* has a mutation in the structural PRPH2 protein. Apart from other potential effects of this mutation difference, it is important to note that the PRPH2 mutation prevents the *rd2* photoreceptors from forming outer segments [39]. This most likely also affects events in the inner segments, such as rhodopsin production, as active rhodopsin is not measurable in the *rd2* retina [39]. As EPAC2 is expressed in the inner segments, the lack of outer segments in *rd2* could thus have cellular consequences that stretch as far as to EPAC2 responses in general. Furthermore, it can be suggested that if cGMP can mediate EPAC2 activity in the retina, then due to the lower cGMP level within *rd2* [13,40], cGMP might not influence EPAC2 as much as otherwise seen in the *rd1* and *rd10.*

### 3.3. The EPAC2 Interactors cGMP and cAMP Accumulate during Degeneration 

EPAC2 interacts with both cAMP and cGMP [21,22], so the photoreceptor EPAC2 could be influenced by alterations in the levels of these nucleotides. The compromised PDE6β activity in *rd10* and *rd1* leads to the accumulation of photoreceptor cGMP [13]. Here, we demonstrated that such accumulation is true also for cAMP, in particular for *rd1*. In part, the increased cAMP as such may be the consequence of a potential—activatory or inhibitory—by high cGMP levels acting on the cAMP hydrolyzation through the cGMP-regulated PDEs (PDE2 and/or PDE3) [41]. A dysregulation might also, by feedback, affect cAMP production via adenylyl cyclase, which is known to have a connection to retinal degeneration [42].

It has been suggested that cAMP protects against photoreceptor degeneration in the early stages [35], and although the mechanisms have not been specifically studied, one possibility is that this is brought about by cAMP’s ability to activate EPAC [19]. The *rd1* retina showed both cellular colocalization of EPAC2 and cGMP and increased EPAC2:cGMP interactions with time, but neither of these was seen for cAMP. In fact, the above-reported cAMP level in *rd10* could match the low EPAC2:cAMP interactions in the same model. Although the low EPAC2:cAMP interactions in *rd10* were not significantly different from its wt, they were seemingly lower than the situation in *rd1*. Moreover, the EPAC2 activity was lower in the degenerating *rd10* retina than in healthy wt (EPAC activity was not measured in *rd1*). Altogether, supports an early cGMP and EPAC2 increase in the degenerating photoreceptors, which allows for more EPAC2:cGMP interactions at the expense of cAMP activation of EPAC2. 

### 3.4. Inhibiting EPAC2 Activity Reduces ONL Thickness in the rd10 Model

The inhibitor reduced EPAC2 activity in wt explants, as measured by an Rap1-GTP activity assay, but it also caused a significantly lower EPAC2 expression in the wt ONL. The latter was unexpected because, if anything, a compensatory increase would have been anticipated, and we currently have no explanation for this.

**Figure 7:**
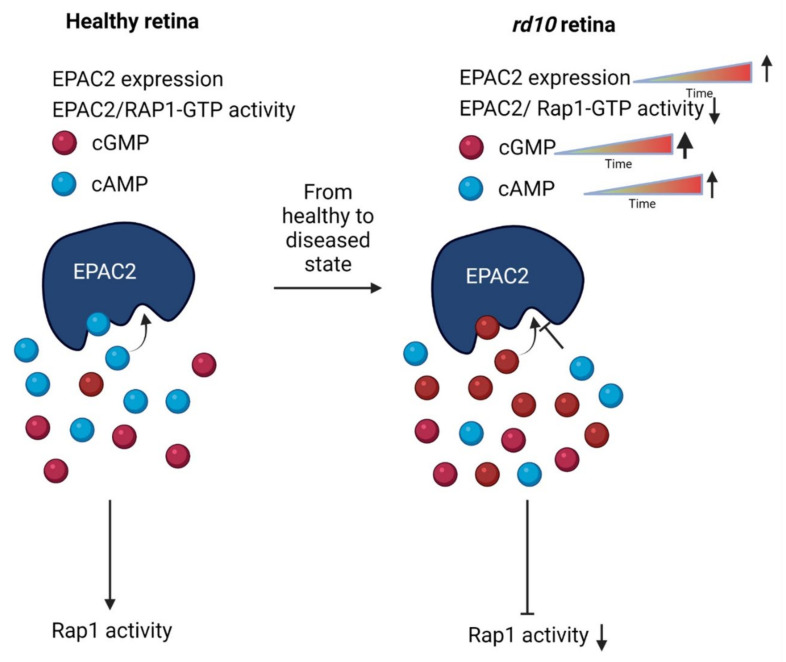
Illustration of cGMP’s suggested effect on EPAC2 activity based on data from both *rd1* and *rd10*. In the *rd10* model, EPAC2 activity is decreased compared to wt. Therefore, as a speculation, the *rd10* model may compensate for the activity loss by expressing more EPAC2 protein over time within the ONL, which was also seen with *rd1*. The decreased EPAC2 activity could be the result of increasing cGMP levels, which compete with cAMP for the binding of EPAC2. Increased cGMP binding might in turn prevent stimulation of protective pathways, even if the cAMP level would subsequently increase. The up and down arrows indicate an increase or decrease, respectively, of either Rap1 activity or EPAC2 expression.

During EPAC2 inhibition in the *rd10* explants, there was no significant effect on EPAC2 expression. However, the ONL thickness was reduced, which is likely the result of increased photoreceptor cell death. Further studies may reveal whether different EPAC2 activation paradigms instead will have an opposite effect and prevent such cell death. The lack of effect on TUNEL of the inhibitor could be due to the fact that the peak of *rd10* TUNEL positivity had already passed by several days at the time of analysis [13]. The peak of TUNEL positivity for a particular photoreceptor is most likely to last less than 10 h [12], and the absence of accumulated differences at any given time point would then make detection of smaller effects less likely. The outcome suggests that inhibition of EPAC2 activity is detrimental to photoreceptors (Figure 7).

## 4. Materials and Methods

### 4.1. Materials

All chemicals were commercially available. Protease inhibitor (cat no. P5726), phosphatase inhibitor (cat no. P3840), and TUNEL assay (cat. no. 12156792910) were from Sigma (St. Louis, MO, USA). The Bio-Rad Protein Assay Kit (cat. no. 5000112) and DL-dithiothreitol (DTT, cat. no. 1610610) were from Bio-Rad (Munich, Germany). PageBlue protein staining solution (cat. no. 24620), Nupage gels 4–12% (cat. no. NP0322PK2), NuPage sample buffer (cat. no. NP0008), MOPS buffer (cat. no. B0001), prestained PageRuler (cat. no. 26616), and Pierce ECL Western blotting substrate (cat. no. 32106) were from Thermo Scientific (Waltham, MA, USA). The recombinant EPAC2 (ab153155) protein was purchased from Abcam (Boston, MA, USA). EPAC2 activator (S-220; cat. no. B 046) and EPAC2 inhibitor (ESI-05; cat. no. M 092) were purchased from BIOLOG Life Science Institute (Bremen, Germany). All utilized antibodies and dilutions are listed in Table 1. 

### 4.2. Animals

Animals used were the following three different *rd* mouse lines—C3H rd1/rd1 (rd1 [44]), C57BL/6J rd10/rd10 (rd10, RRID:MGI:3581193, The Jackson Laboratory, Bar Harbor, ME, USA), and C3H rd2/rd2 (rd2 [45])—as well as the healthy counterpart wild-type C3H [44]. No randomization of mice nor any sample calculation was performed in this study. All lines are kept and bred in-house. The animals were housed under standard white cyclic lighting, had free access to food and water, and were used irrespective of sex. All the procedures were performed following the issued local animal ethics committee (permit #M92-15 and 02124/2020) and the ARVO statement for the use of animals in ophthalmic and visual research. All efforts were made to minimize the number of animals used and their suffering. Prior to eye enucleation, the mice were sacrificed as required by the permit. The study was not preregistered. 

### 4.3. Organotypic Retinal Explant Culture

Organotypic retinal cultures derived from *rd10* and wt animals were prepared under sterile conditions as previously described [46,47]. Animals at postnatal day 12 (PN12) were sacrificed, and eyes were enucleated and incubated in R16 basal medium (07491252A; Gibco) with 1.2% proteinase K (10103533; Thermo Fisher, Waltham, MA, USA) for 15 min at 37 °C to separate the retinal pigment epithelium (RPE) from the sclera. This was followed by adding 10% fetal bovine serum (10082139; Thermo Fisher) plus rinsing in R16 medium to block proteinase K activity. The retina with attached RPE was flat-mounted on a culture membrane insert (PIHP03050; Merk Millipore, Burlington, MA, USA) with the RPE facing the membrane. The membrane inserts were plated in six-well culture plates and incubated with R16 culture medium complemented with supplements and free of serum and antibiotics in a humidified incubator (5% CO2) at 37 °C. For the first 48 h, the retinas were left undisturbed to adapt to the culturing conditions. They were then treated with either EPAC2 activator (S-220) or inhibitor (ESI-05) at 50 µM or kept as untreated controls; the latter was given a similar dilution solvent (DMSO was used to dissolve the S-220 and ESI-05) volume. In both cases, the medium, including the additions, was changed every two days. The culture paradigm used was from PN12 to PN24. The culture was terminated with 2 h of fixation in 4% paraformaldehyde (PFA), cryoprotected with graded sucrose solutions containing 10% sucrose followed by 25% sucrose, and then embedded in a BSA-based embedment solution (A2153-100G; Sigma, St. Louis, MO, USA). Tissue sections of 12 µm were prepared using a Thermo Scientific NX50 microtome (Thermo Scientific, Waltham, MA, USA) and thawed on Superfrost Plus slides (R. Langerin, Emmendingen, Germany). 

### 4.4. Immunofluorescence

Retinal cryosections obtained from all *rd* or wt mice were rehydrated in PBS and pre-incubated for 1 h at 22 °C in blocking solution (1% BSA, 0.25% Triton in PBS). Sections were then incubated with primary antibodies overnight at 4 °C (antibodies and dilutions are given in Table 1) and rinsed in PBS. Corresponding secondary antibodies labeled with Alexa fluor 488 or Texas red fluorescent dye (Invitrogen, Carlsbad, CA, USA and Abcam, Biomedical Campus, Cambridge, UK) were added for 45 min at 22 °C. After rinsing, the tissues were mounted in Flouroshield mounting medium with DAPI (Abcam, Biomedical Campus, Cambridge, UK) for nuclear counterstaining. For visualization of dying photoreceptors on sections of cultured retinas, a TUNEL assay kit conjugated with TMR red (Roche Diagnostic, Basel, Schweiz) was applied according to the manufacturer’s instructions. For the intensity measurements, all stainings at the respective ages (i.e., wt:*rd1,* wt:*rd10*, and wt:*rd2*) were performed in pairs (wt + model) in an identical fashion (i.e., same pAb and sAb solutions and incubation times). Furthermore, images were taken using identical microscope settings using Zen (2.5) blue edition software (Zeiss Zen 3.3 software). The immunofluorescence intensity, outer nuclear layer (ONL) thickness, and TUNEL-positive cells were then quantified by first outlining the ONL area using DAPI as a reference, after which the respective values were quantified using GraphPad Prism 8. The choice of using fluorescent intensity rather than Western blotting for quantifying, e.g., EPAC2 expression, was based on the questions we wanted to ask. Because we were interested in separating data from, for instance, ONL and segments, we needed an approach that allows this in a strict and repeatable fashion, which excluded Western blotting. In the latter, it is not possible to isolate pure photoreceptors or their segments without the risk of losing important parts of the desired area or of contaminating the samples with other retinal cells—both of which would result in a false output. 

For the in vivo experiments, each time point represents between three and seven biologically independent sections, whereas for the organotypic retinal explants, each data point represents three to six replicates. All samples were quantified using Zen (2.5) blue edition software (Zeiss Zen software). The area of interest was measured by defining the segments, ONL, and INL, and the latter two were highlighted with the aid of DAPI staining. Segments were identified above the ONL, and the fluorescent intensity from the EPAC2 expression within these marked areas was then quantified by the software. For multiple statistical comparisons between the respective *rd* and wt and their different layers, as well as EPAC2 expression within the ONL at different time points, a Kruskal–Wallis or two-way ANOVA test with the Holm–Sidak correction was applied. The test was performed using GraphPad Prism 8. Error bars in figures represent SD. Levels of significance are * *p* < 0.05, ** *p* < 0.01, *** *p* < 0.001, and **** *p* < 0.0001. Fluorescent images displayed within this report have been adjusted with respect to the fluorescent intensity to improve their clarity and visibility.

### 4.5. Proximity Ligation Assay (PLA)

PLA was performed on cryosections of 4% PFA-fixed retinas using a Detection Reagents Red kit (cat no. DUO92008; Sigma, St. Louis, MO, USA); the PLA Probe anti-rabbit or anti-goat MINUS; and the antibodies cGMP, cAMP (for antibody specificity; see Figure S1), or EPAC2 (for antibody specificity; see Figure S2). The cGMP and cAMP antibodies had a PLUS probe, and the EPAC2 had a MINUS probe attached using Duolink^®^ In Situ Probemaker PLUS (cat no. DUO92009; Sigma, St. Louis, MO, USA) or MINUS (cat no. DUO92010; Sigma, St. Louis, MO, USA). All PLA reagents were from Duolink (Olink Bioscience, Uppsala, Sweden). The procedure followed the manufacturer’s instructions. In short, the retinal sections were permeabilized and blocked with a blocking solution (1% BSA, 0.25% Triton X-100 in PBS) for 45 min at 22 °C, and primary antibodies were incubated overnight at 4 °C. The ligation and amplification steps were performed using the Detection Reagents Red kit reagents, followed by the addition of a mounting medium with DAPI. To visualize the PLA punctuations, pictures were taken of the whole retinal cross-sections at 20x magnification using a Zeiss Axio Imager M2 microscope (Carl Zeiss, Oberkochen, Germany). The total number of PLA punctuations and area of the ONL in the images from samples from three to six biological replicas were quantified with the aid of Zen (2.5) blue edition software (Zeiss Zen software). The PLA images had not been made unidentifiable prior to quantification. To consider the decreasing ONL area and avoid bias due to degeneration, the results were depicted with histograms as the ratio of PLA punctuations over the area (µm^2^) in the ONL using GraphPad Prism 8.1. 

### 4.6. Rap1-GTP Activity Assay

Retinas from wt and *rd10* (PN17, wt = 16 retina; *rd10* = 15 retina), as well as cultured wt (PN17, Ctrl = 10 retina; S-220 = 10 retina; ESI-05 = 11 retina) retinas that had been pharmacologically manipulated for 1 h with either S-220 or ESI-05 or kept untreated (Ctrl), were collected and snap-frozen until further use. The Rap1-GTP assay was conducted as described by the manufacturer, with a few modifications to avoid sample loss. This included that the retinas were times kept on ice at all times and lysed with ice-cold lysis buffer, including phosphatase and protease inhibitor, followed by centrifugation at 6000× *g* for 15 min at 4 °C. The soluble fraction’s protein concentration was measured by the Bio-Rad protein assay kit. The affinity precipitation of activated Rap1 was prepared by adding 100 µL glutathione resin to a tube, followed by centrifugation at 6000× *g* for 1 min at 4 °C to discard the liquid from the resins. The resins were then washed with lysis buffer, and the buffer was removed after centrifugation, as described above. GST-RaIGDS-RBD (20 µg) was added to the resins together with a 1.5 mg retinal sample per condition. The samples were left to incubate for 1 h at 4 °C under rotation, after which the resins were washed with lysis buffer thrice before adding 25 µL loading buffer to the resins and heating the samples at 95 °C for 5 min. After adding 25 µL of the isolated activated Rap1 to the gels, 20 µg of raw lysate from the original samples was also loaded to the gel. Western blot was performed as previously mentioned; the primary antibody Rap1 (ab75871, Abcam) was used to detect the protein at 21 kDa. The Rap1 band’s intensity was normalized to the Coomassie-blue-stained gels before the Rap1-GTP was normalized to the total amount of Rap1 in the samples. It should be noted that the Rap1-GTP activity assay was only performed once per sample type due to the large amount of retinas required to obtain a signal. Furthermore, we applied the whole retina, even if this meant that we lost the determination of various layers. The assay requires homogenization of tissue samples, so the use of histological sections like in the EPAC2 measurement was not possible, nor was it possible to isolate pure photoreceptors without the risk of loss or contaminating the samples with other retinal cells. It is therefore important to notice that this assay reflects the total Rap1-GTP activity within the whole retina. 

## 5. Conclusions

We used immunostainings and organotypic retinal cultures in combination with well-established *rd* models (i.e., *rd1*, *rd10*, and *rd2*), as well as their respective healthy counterparts, to investigate the involvement of EPAC2 in degenerating photoreceptors. The results showed increased EPAC2 expression with time in the *rd1* and *rd10* models and increased EPAC2:cGMP interactions in *rd1*. In turn, the latter prevented EPAC2, which is normally activated by cAMP binding, from exerting photoreceptor-protective actions (Figure 7). Prevention of cGMP binding to EPAC2 may thus, after further exploration, become an interesting novel route to photoreceptor protection. 

## Figures and Tables

**Table 1:** Antibody list.

Antibody	Provider	Cat. No.	Dilution IF/PLA
Sheep anti-cGMP	Provided by Harry W.M. Steinbusch, Maastrich University, the Netherlands [43]	Not applicable	1:500
Rabbit anti-EPAC2	Sigma (St. Louis, MO, USA)	ABN492	1:300
Mouse-anti Rhodopsin	Sigma (St. Louis, MO, USA)	Mab5316	1:300
Rabbit-anti cAMP	Sigma (St. Louis, MO, USA)	07-1497	1:300
Lectin PNA Alexa Fluor™ 594 Conjugate	Thermo Scientific (Waltham, MA, USA)	L32459	1:1000
Mouse-anti ATP1A3	Abcam (Cambridge, UK)	Ab2826	1:300

## Data Availability

The data presented in this study are available in Supplementary Figures S1–S6.

## Supplementary Materials

The following supporting information can be downloaded at: https://www.mdpi.com/article/10.3390/ijms23094619/s1.

## References

[1] Fahim A.T., Daiger S.P., Weleber R.G. Nonsyndromic Retinitis Pigmentosa Overview. GeneReviews^®^. http://www.ncbi.nlm.nih.gov/pubmed/20301590.

[2] Chang S., Vaccarella L., Olatunji S., Cebulla C., Christoforidis J. (2011). Diagnostic Challenges in Retinitis Pigmentosa: Genotypic Multiplicity and Phenotypic Variability. Curr. Genom..

[3] Pfeiffer R.L., Marc R.E., Jones B.W. (2020). Persistent remodeling and neurodegeneration in late-stage retinal degeneration. Prog. Retin. Eye Res..

[4] Hanna J., Yücel Y.H., Zhou X., Mathieu E., Paczka-Giorgi L.A., Gupta N. (2017). Progressive loss of retinal blood vessels in a live model of retinitis pigmentosa. Can. J. Ophthalmol..

[5] Petit L., Lhériteau E., Weber M., Le Meur G., Deschamps J.Y., Provost N., Mendes-Madeira A., Libeau L., Guihal C., Colle M.A. (2012). Restoration of vision in the PDE6β-deficient dog, a large animal model of rod-cone dystrophy. Mol. Ther..

[6] Haim M. (2002). The epidemiology of retinitis pigmentosa in Denmark. Acta Ophthalmol. Scand. Suppl..

[7] Pichard V., Provost N., Mendes-Madeira A., Libeau L., Hulin P., Tshilenge K.T., Biget M., Ameline B., Deschamps J.Y., Weber M. (2016). AAV-mediated gene therapy halts retinal degeneration in PDE6β-deficient dogs. Mol. Ther..

[8] Russell S., Bennett J., Wellman J.A., Chung D.C., Yu Z.F., Tillman A., Wittes J., Pappas J., Elci O., McCague S. (2017). Efficacy and safety of voretigene neparvovec (AAV2-hRPE65v2) in patients with RPE65-mediated inherited retinal dystrophy: A randomised, controlled, open-label, phase 3 trial. Lancet.

[9] Power M., Das S., Schütze K., Marigo V., Ekström P., Paquet-Durand F. (2020). Cellular mechanisms of hereditary photoreceptor degeneration—Focus on cGMP. Prog. Retin. Eye Res..

[10] Farber D.B., Lolley R.N. (1974). Cyclic guanosine monophosphate: Elevation in degenerating photoreceptor cells of the C3H mouse retina. Science.

[11] Paquet-Durand F., Hauck S.M., Van Veen T., Ueffing M., Ekström P. (2009). PKG activity causes photoreceptor cell death in two retinitis pigmentosa models. J. Neurochem..

[12] Sahaboglu A., Paquet-Durand O., Dietter J., Dengler K., Bernhard-Kurz S., Ekström P.A.R., Hitzmann B., Ueffing M., Paquet-Durand F. (2013). Retinitis pigmentosa: Rapid neurodegeneration is governed by slow cell death mechanisms. Cell Death Dis..

[13] Arango-Gonzalez B., Trifunović D., Sahaboglu A., Kranz K., Michalakis S., Farinelli P., Koch S., Koch F., Cottet S., Janssen-Bienhold U. (2014). Identification of a common non-apoptotic cell death mechanism in hereditary retinal degeneration. PLoS ONE.

[14] Corradini E., Burgers P.P., Plank M., Heck A.J.R., Scholten A. (2015). Huntingtin-Associated Protein 1 (HAP1) is a cGMP-dependent Kinase Anchoring Protein (GKAP) specific for the cGMP-dependent protein kinase Iβ isoform. J. Biol. Chem..

[15] Francis S.H., Corbin J.D. (2013). Cyclic Nucleotide-Dependent Protein Kinases. Encycl. Biol. Chem. Second Ed..

[16] Francis S.H., Blount M.A., Zoraghi R., Corbin J.D. (2005). Molecular properties of mammalian proteins that interact with cGMP: Protein kinases, cation channels, phosphodiesterases, and multi-drug anion transporters. Front. Biosci..

[17] Mazzolini M., Arcangeletti M., Marchesi A., Napolitano L.M.R., Grosa D., Maity S., Anselmi C., Torre V. (2018). The gating mechanism in cyclic nucleotide-gated ion channels. Sci. Rep..

[18] Vighi E., Trifunović D., Veiga-Crespo P., Rentsch A., Hoffmann D., Sahaboglu A., Strasser T., Kulkarni M., Bertolotti E., Van Den Heuvel A. (2018). Combination of cGMP analogue and drug delivery system provides functional protection in hereditary retinal degeneration. Proc. Natl. Acad. Sci. USA.

[19] Rehmann H., Arias-Palomo E., Hadders M.A., Schwede F., Llorca O., Bos J.L. (2008). Structure of Epac2 in complex with a cyclic AMP analogue and RAP1B. Nature.

[20] Rasmussen M., Welinder C., Schwede F., Ekström P. (2020). The cGMP system in normal and degenerating mouse neuroretina: New proteins with cGMP interaction potential identified by a proteomics approach. J. Neurochem..

[21] Rehmann H., Schwede F., Doøskeland S.O., Wittinghofer A., Bos J.L. (2003). Ligand-mediated activation of the cAMP-responsive guanine nucleotide exchange factor Epac. J. Biol. Chem..

[22] Christensen A.E., Selheim F., De Rooij J., Dremier S., Schwede F., Dao K.K., Martinez A., Maenhaut C., Bos J.L., Genieser H.G. (2003). cAMP analog mapping of Epac1 and cAMP kinase: Discriminating analogs demonstrate that Epac and cAMP kinase act synergistically to promote PC-12 cell neurite extension. J. Biol. Chem..

[23] Liu C., Takahashi M., Li Y., Dillon T.J., Kaech S., Stork P.J.S. (2010). The Interaction of Epac1 and Ran Promotes Rap1 Activation at the Nuclear Envelope. Mol. Cell. Biol..

[24] Suzuki S., Yokoyama U., Abe T., Kiyonari H., Yamashita N., Kato Y., Kurotani R., Sato M., Okumura S., Ishikawa Y. (2010). Differential roles of Epac in regulating cell death in neuronal and myocardial cells. J. Biol. Chem..

[25] Shim M.S., Kim K.Y., Bu J.H., Nam H.S., Jeong S.W., Park T.L., Ellisman M.H., Weinreb R.N., Ju W.K. (2018). Elevated intracellular cAMP exacerbates vulnerability to oxidative stress in optic nerve head astrocytes article. Cell Death Dis..

[26] Das R., Chowdhury S., Mazhab-Jafari M.T., SilDas S., Selvaratnam R., Melacini G. (2009). Dynamically driven ligand selectivity in cyclic nucleotide binding domains. J. Biol. Chem..

[27] Whitaker C.M., Cooper N.G.F. (2010). Differential distribution of exchange proteins directly activated by cyclic AMP within the adult rat retina. Neuroscience.

[28] Liu W., Ha Y., Xia F., Zhu S., Li Y., Shi S., Mei F.C., Merkley K., Vizzeri G., Motamedi M. (2020). Neuronal Epac1 mediates retinal neurodegeneration in mouse models of ocular hypertension. J. Exp. Med..

[29] Ramos C.J., Lin C., Liu X., Antonetti D.A. (2018). The EPAC-Rap1 pathway prevents and reverses cytokineinduced retinal vascular permeability. J. Biol. Chem..

[30] Zhang L., Zhang L., Liu H., Jiang F., Wang H., Li D., Gao R. (2018). Inhibition of Epac2 Attenuates Neural Cell Apoptosis and Improves Neurological Deficits in a Rat Model of Traumatic Brain Injury. Front. Neurosci..

[31] Guijarro-Belmar A., Domanski D.M., Bo X., Shewan D., Huang W. (2021). The therapeutic potential of targeting exchange protein directly activated by cyclic adenosine 3′,5′-monophosphate (Epac) for central nervous system trauma. Neural Regen. Res..

[32] Blanks J.C., Johnson L.V. (1984). Specific binding of peanut lectin to a class of retinal photoreceptor cells. A species comparison. IOVS.

[33] Johnson L.V., Hageman G.S., Blanks J.C. (1986). Interphotoreceptor matrix domains ensheath vertebrate cone photoreceptor cells. IOVS.

[34] Molday L.L., Cheng C.L., Molday R.S. (2019). Cell-Specific Markers for the Identification of Retinal Cells and Subcellular Organelles by Immunofluorescence Microscopy. Methods Mol. Biol..

[35] Traverso V., Bush R.A., Sieving P.A., Deretic D. (2002). Retinal cAMP levels during the progression of retinal degeneration in rhodopsin P23H and S334ter transgenic rats. Investig. Ophthalmol. Vis. Sci..

[36] Bowes C., Li T., Danciger M., Baxter L.C., Applebury M.L., Farber D.B. (1990). Retinal degeneration in the rd mouse is caused by a defect in the β subunit of rod cGMP-phosphodiesterase. Nature.

[37] Carter-Dawson L.D., LaVail M.M., Sidman R.L. (1978). Differential effect of the rd mutation on rods and cones in the mouse retina. Investig. Ophthalmol. Vis. Sci..

[38] Chang B., Hawes N.L., Pardue M.T., German A.M., Hurd R.E., Davisson M.T., Nusinowitz S., Rengarajan K., Boyd A.P., Sidney S.S. (2007). Two mouse retinal degenerations caused by missense mutations in the β-subunit of rod cGMP phosphodiesterase gene. Vision Res..

[39] Sanyal S., Jansen H.G. (1981). Absence of receptor outer segments in the retina of rds mutant mice. Neurosci. Lett..

[40] Wang T., Tsang S.H., Chen J. (2017). Two pathways of rod photoreceptor cell death induced by elevated cGMP. Hum. Mol. Genet..

[41] Yan C., Miller C.L., Abe J.I. (2007). Regulation of Phosphodiesterase 3 and Inducible cAMP Early Repressor in the Heart. Circ. Res..

[42] Nakao T., Tsujikawa M., Notomi S., Ikeda Y., Nishida K. (2012). The Role of Mislocalized Phototransduction in Photoreceptor Cell Death of Retinitis Pigmentosa. PLoS ONE.

[43] Tanaka J., Van Ittersum M.M., Steinbusch H.W.M., De Vente J. (1997). Nitric oxide-mediated cGMP synthesis in oligodendrocytes in the developing rat brain. Glia.

[44] Sanyal S., Bal A.K. (1973). Comparative light and electron microscopic study of retinal histogenesis in normal and rd mutant mice. Z. Anat. Entwicklungsgesch..

[45] Sanyal S., Zeilmaker G.H. (1984). Development and degeneration of retina in rds mutant mice: Light and electron microscopic observations in experimental chimaeras. Exp. Eye Res..

[46] Belhadj S., Tolone A., Christensen G., Das S., Chen Y., Paquet-Durand F. (2020). Long-Term, Serum-Free Cultivation of Organotypic Mouse Retina Explants with Intact Retinal Pigment Epithelium. JoVE J. Vis. Exp..

[47] Caffé A.R., Ahuja P., Holmqvist B., Azadi S., Forsell J., Holmqvist I., Söderpalm A.K., Van Veen T. (2002). Mouse retina explants after long-term culture in serum free medium. J. Chem. Neuroanat..

