# Supplementary material for: Enhanced cGMP Interactor Rap Guanine Exchange Factor 4 (EPAC2) Expression and Activity in Degenerating Photoreceptors: A Neuroprotective Response?
Source: Int J Mol Sci. 2022 Apr 21;23(9):4619. doi: 10.3390/ijms23094619 (PMC9103912; doi:10.3390/ijms23094619)
Supplement: Supplementary file 1 [file ijms-23-04619-s001.zip › ijms-1629758-supplementary.pdf]

Figure S1

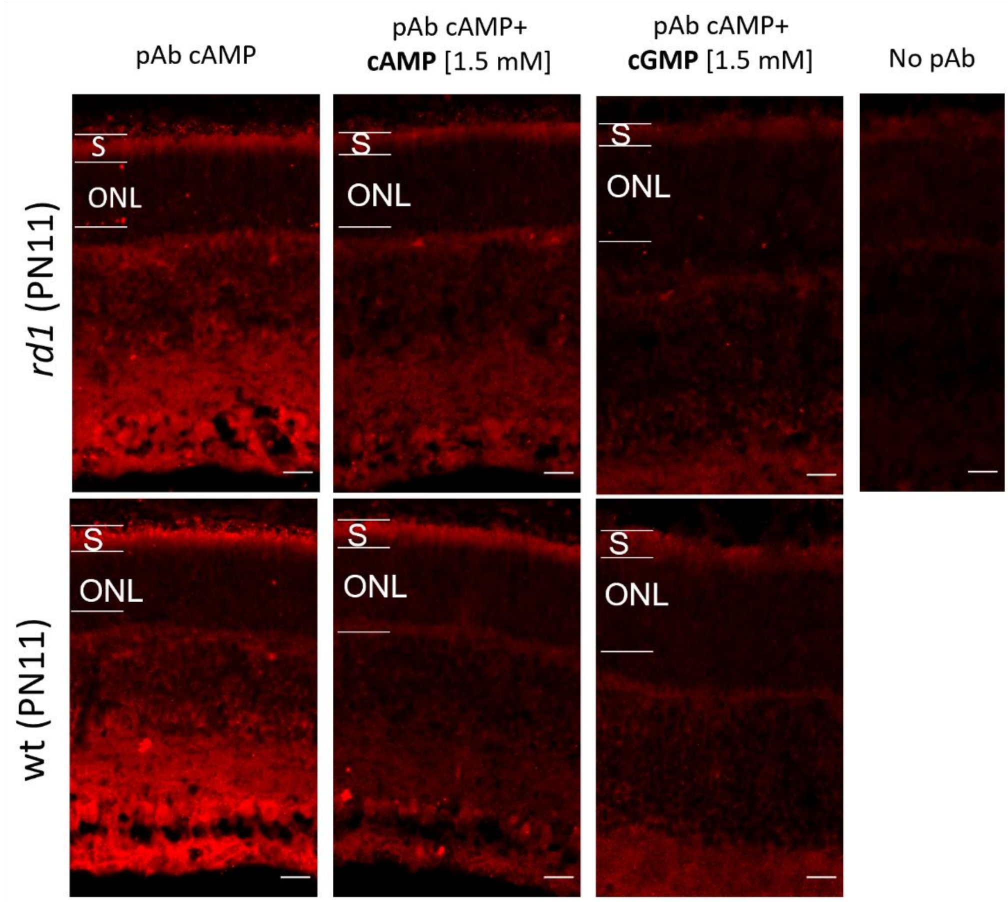

Figure S1 I The cAMP antibody shows selectivity. *Immunostaining showing the selectivity of the cAMP antibody which was tested by pre-incubation of the antibody with either free cAMP [1.5 mM] or cGMP [1.5 mM] prior to addition to samples. Here free cAMP was able to compete with the cAMP for the cAMP antibody in the retinal samples in wt and* rd1 *(PN11). Within the ONL small punctuations are observed suggesting accumulated cAMP, and these are not present in the pAb cAMP+cAMP 1.5 mM* rd1 *section. Free cGMP was not able to compete in this way. The figure represents three biological replicates per staining. S: segments, ONL: outer nuclear layer. Scale bar: 50 µm.*

Figure S2

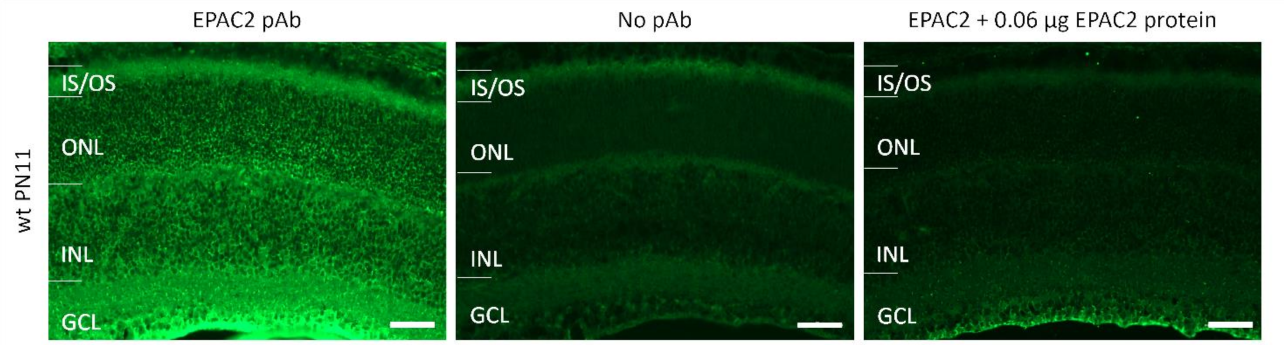

Figure S2 I The EPAC2 antibody shows specificity. *Immunostaining of wt PN11 reitna showing the specificity of the primary EPAC2 antibody which was tested by pre-incubation of the antibody with free EPAC2 protein [0.06 µg] prior addition to retinal sections. Here free EPAC2 was able to compete with the EPAC2 antibody. When no primary EPAC2 antibody (No pAb) was added no fluorescent signal was observed. The EPAC2 expression was found within the IS/OS: segments, ONL: outer nuclear layer, INL: inner nuclear layer as well the GCL: ganglion cell layer. The pictures represent three biological replicates per. staining. Scale bar: 50 µm.*

Figure S3

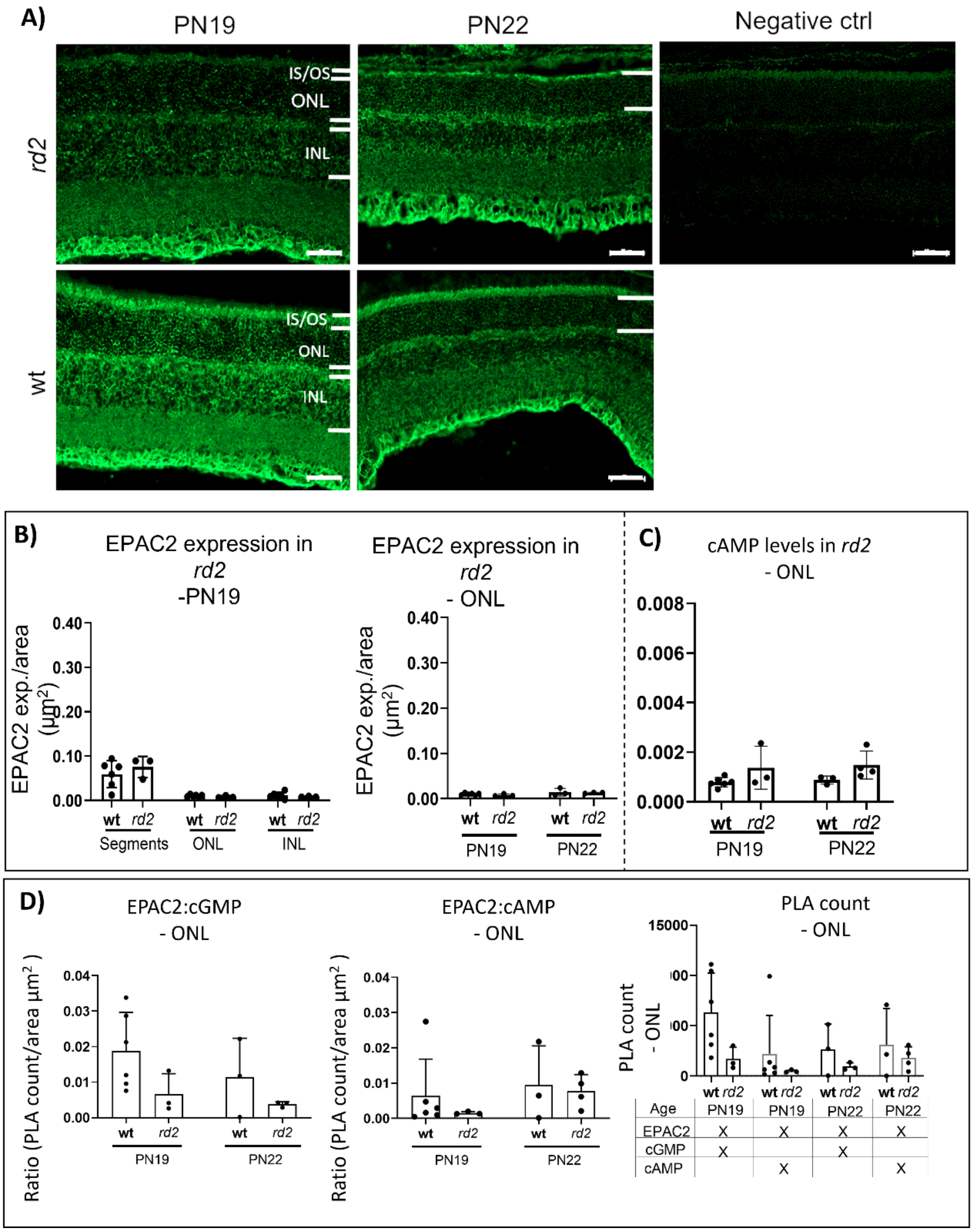

Figure S3 I EPAC2 expression in **rd2** ***and wt retina.*** *A-B) The EPAC2 expression within the ONL in the* rd2 *model does not increase over time and remains similar to the wt retina. C) The cAMP level within the* rd2 *does not change with time. D) For the* rd2 *model, both EPAC2:cGMP and EPAC2:cAMP seem lower in the* rd2 *compared to its wt counterpart, albeit no significance was detected. The pictures represent 3-6 biological independent replicates. IS/OS: segments, ONL: outer nuclear layer, INL: inner nuclear layer. Scale bar: 50 µm. Graphs represent the following 3-6 biological replicates with mean±SD, the statistical method 2-way ANOVA was applied, but no significance was detected.*

Figure S4

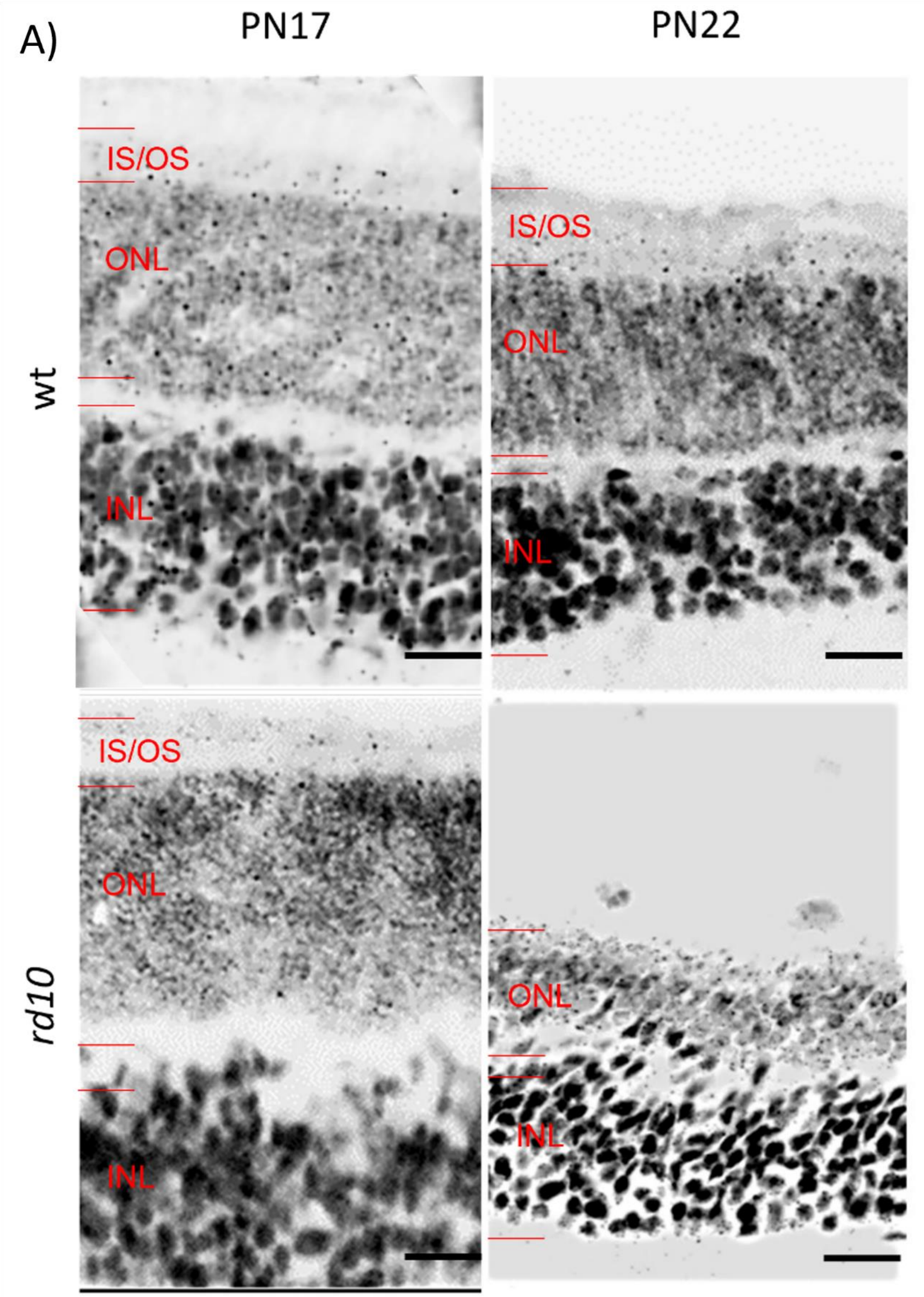
A)
PN17
PN22
wt
rd10
IS/OS
ONL
INL
IS/OS
ONL
INL
IS/OS
ONL
INL
ONL
INL

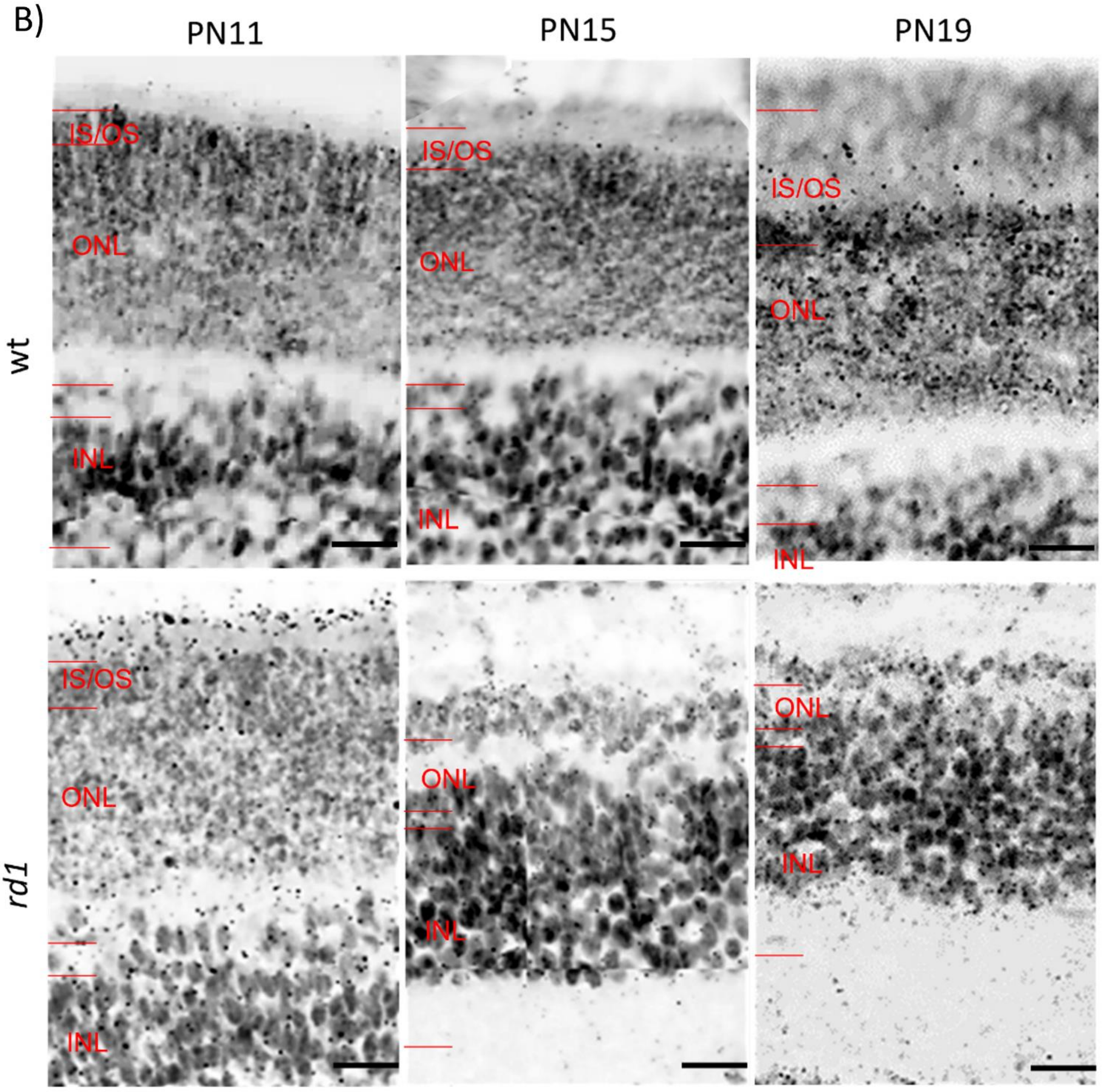
B)
PN11
PN15
PN19
wt
rd1
IS/OS
ONL
INL
IS/OS
ONL
INL
IS/OS
ONL
INL
IS/OS
ONL
INL
ONL
INL
ONL
INL

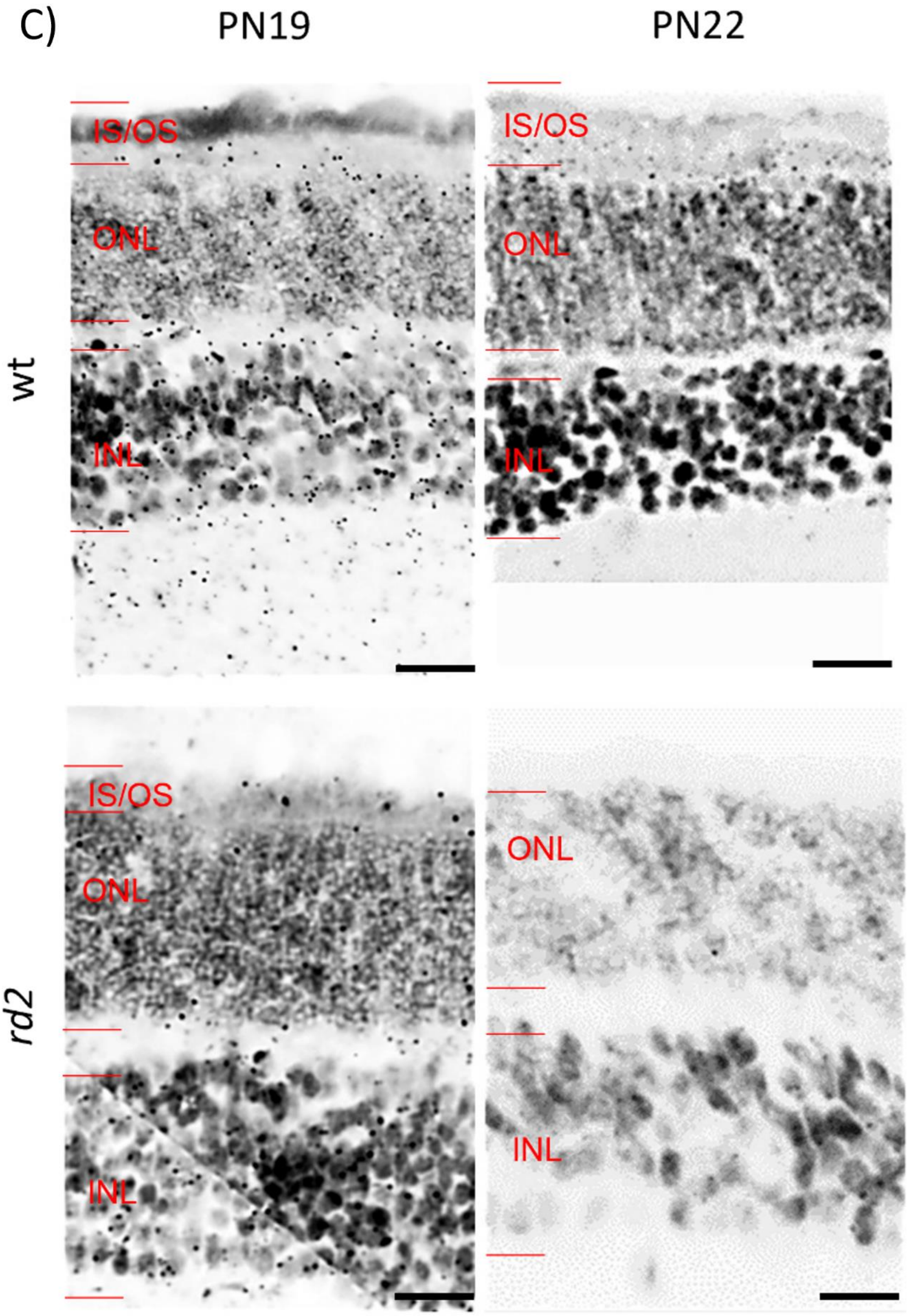
C)
PN19
PN22
wt
rd2
IS/OS
ONL
INL
IS/OS
ONL
INL
IS/OS
ONL
INL
ONL
INL

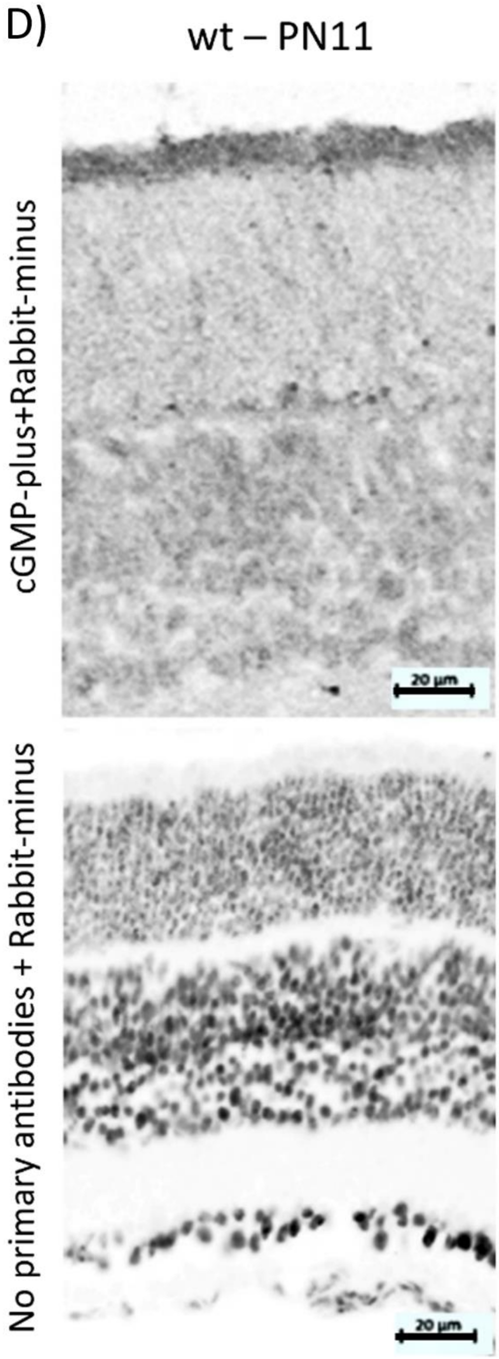

Figure S4 I EPAC2 becomes more in proximity to cGMP in the ONL with time. Magnified sections from the whole retina. *The black PLA punctuations are the positive outcomes from EPAC2:cGMP proximity analyses in* rd *and wt retinas at different ages, respectively. In wt as well as in both* rd *models,* rd10 *(A),* rd1 *(B), and* rd2 *(C). D) The negative controls include cGMP-Plus antibody with anti-rabbit minus-probe (No EPAC2 antibody was added), and no primary antibodies (EPAC2 nor cGMP-PLUS) with anti-rabbit minus. Three biological replicates per staining. IS/OS: segments, ONL: outer nuclear layer, INL: inner nuclear layer. Scale bar: 20 µm.*

Figure S5

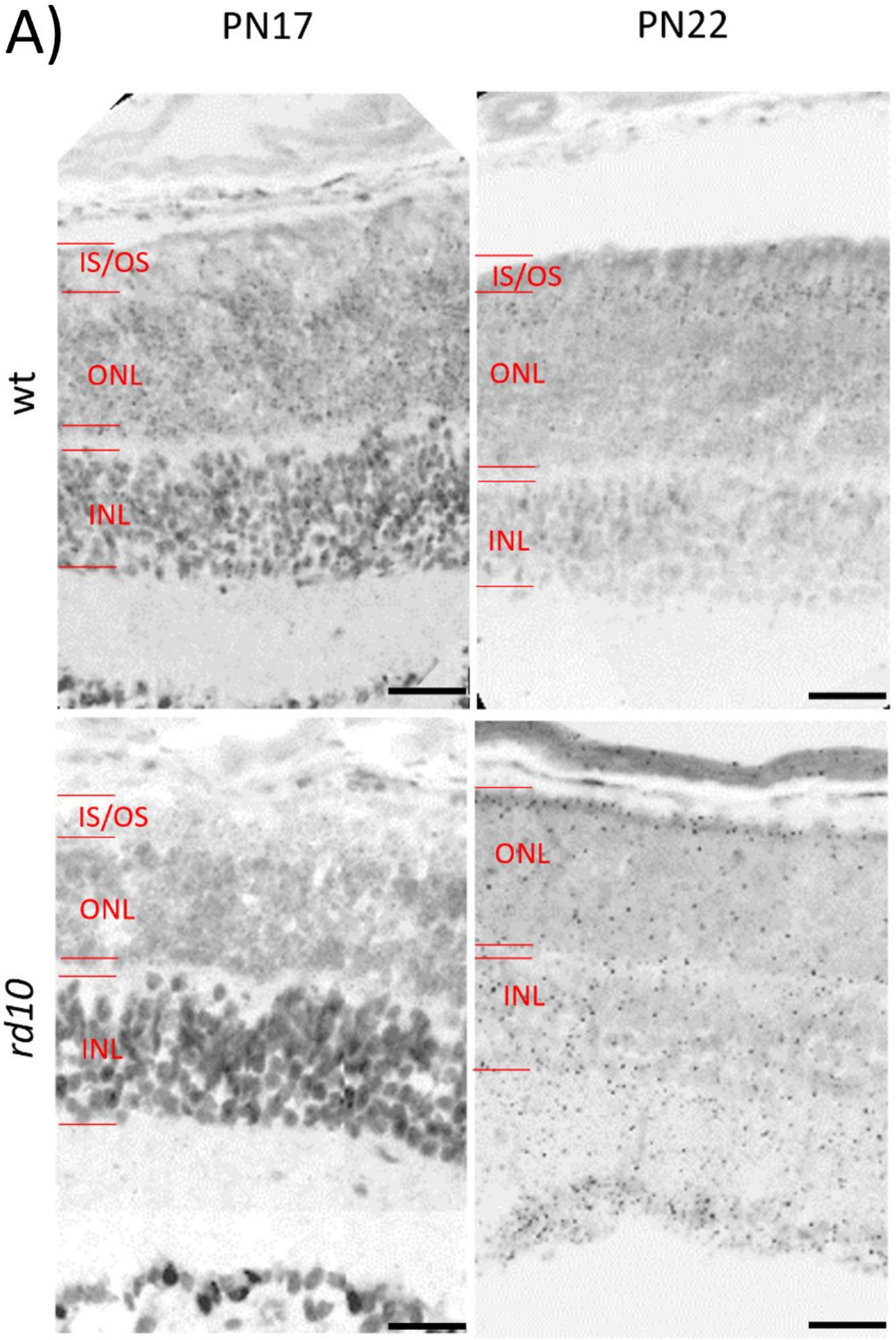
A)
PN17
PN22
wt
rd10
IS/OS
ONL
INL
IS/OS
ONL
INL
IS/OS
ONL
INL
ONL
INL

B)

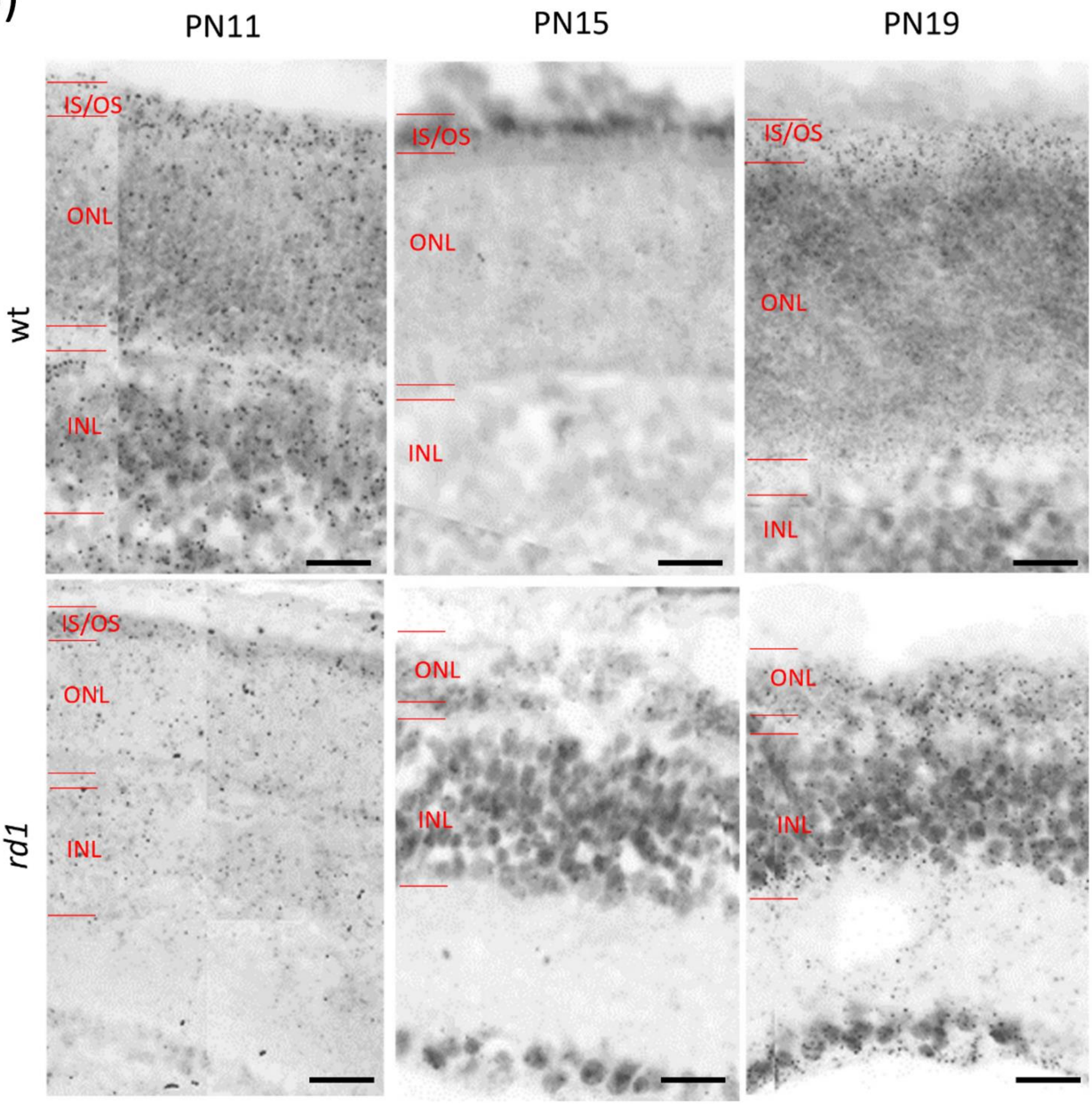
PN11
PN15
PN19
wt
rd1
IS/OS
ONL
INL
IS/OS
ONL
INL
IS/OS
ONL
INL
IS/OS
ONL
INL
ONL
INL
ONL
INL

C)

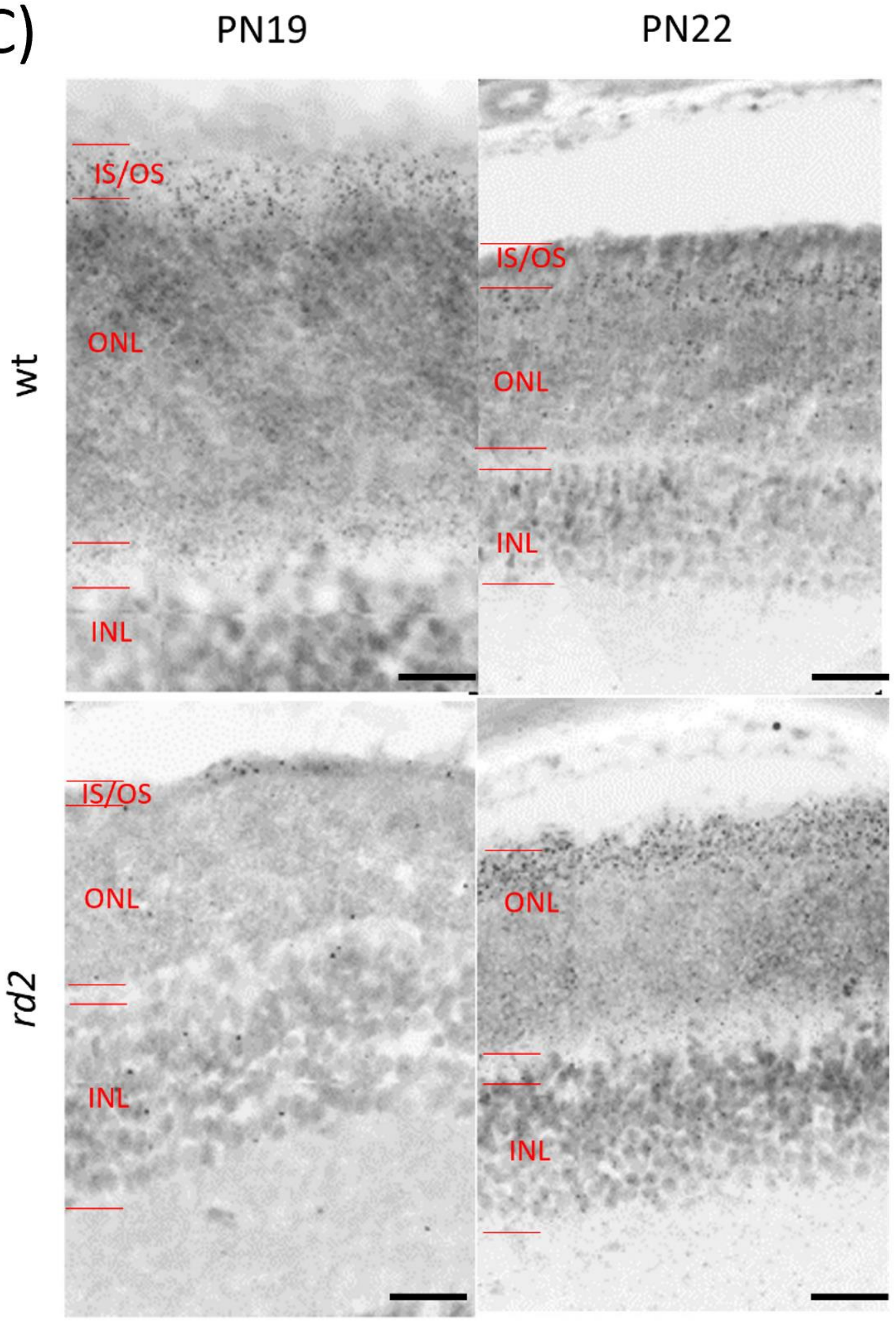

D)

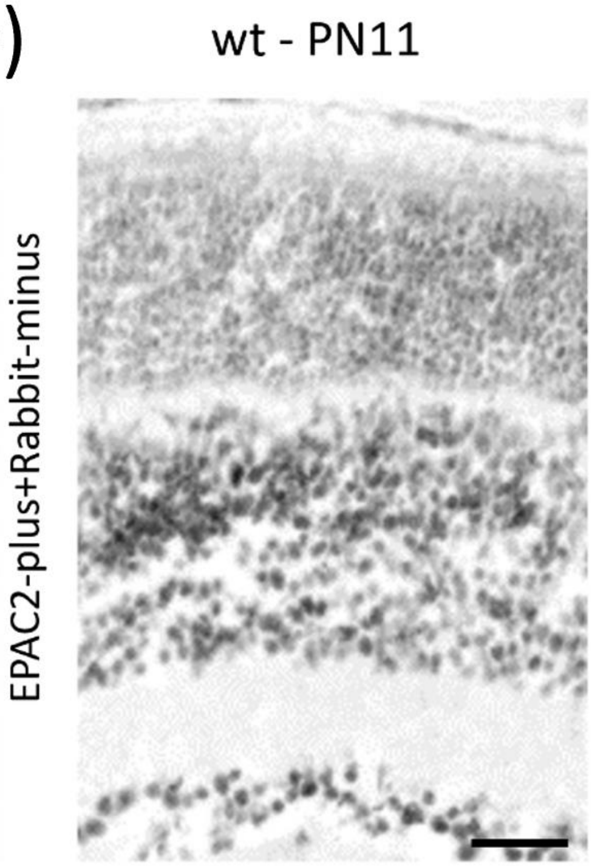

Figure S5 I EPAC2 becomes more in proximity to cAMP with time in the ONL. Magnified sections from the whole retina. *The black punctuations are the positive outcomes from EPAC2:cAMP proximity of the PLA punctuations from* rd *and wt retinas at different ages, respectively. In wt as well as all three* rd *models:* rd10 *(A),* rd1, *(B), and* rd2 *(C). D) The negative Ctrl includes EPAC2-Plus antibody with anti-rabbit minus-probe. Three biological replicates per. staining. IS/OS: segments, ONL: outer nuclear layer, INL: inner nuclear layer. Scale bar: 20 µm.*

Figure S6

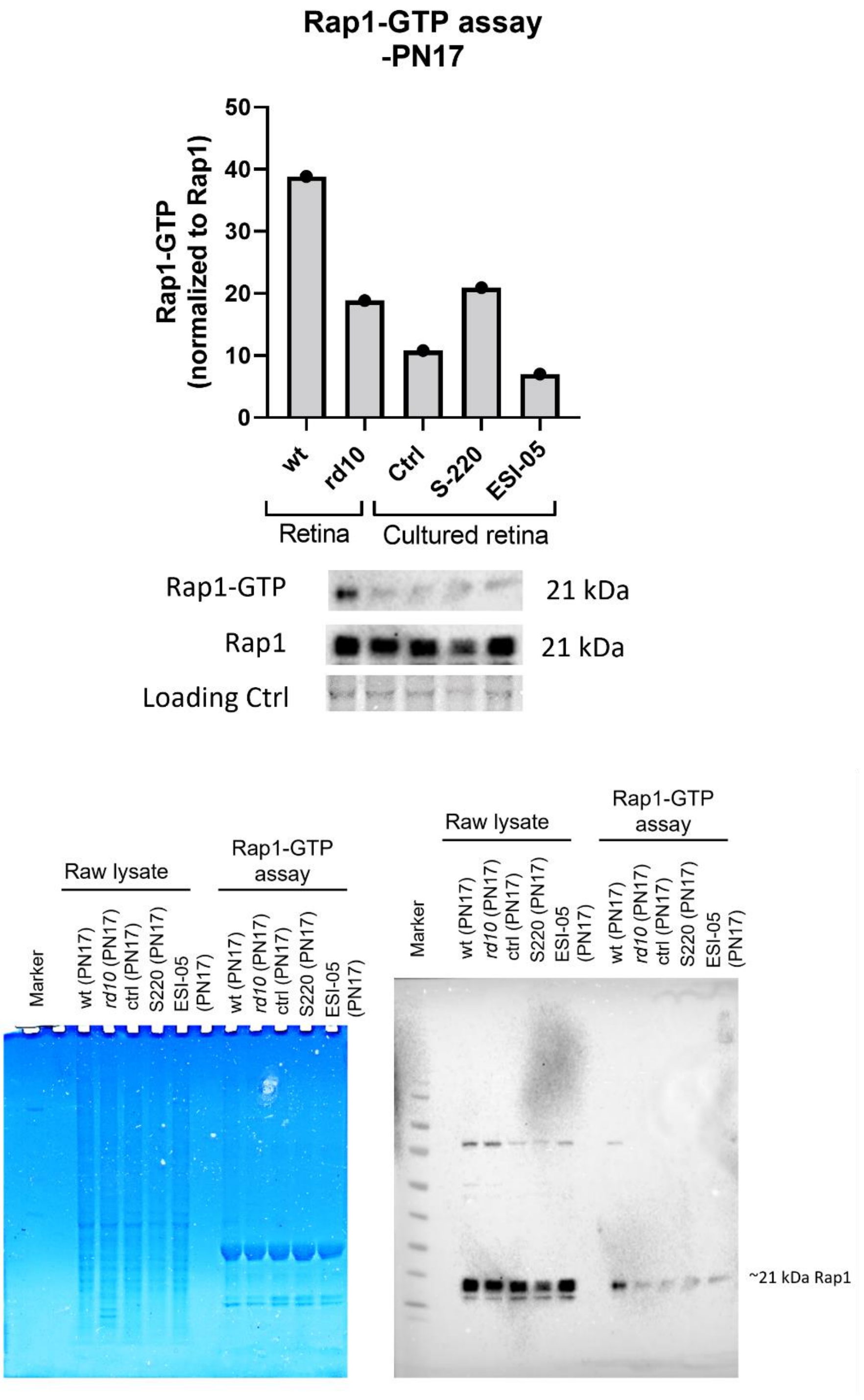

Figure S6 I Rap1-GTP activity assay. *Upper and bottom panel) Measurements of retinas from wt (PN17),* rd10 *(PN17), and cultured wt (PN17) retina pharmacologically manipulated with S-220, ESI-05, or non-treated (i.e., Ctrl) for 1 h, respectively, were subjected to a GTP-assay where Rap1 bound to GTP (i.e., activated Rap1) and was pulled-down. Upper panel) The* rd10 *retina gave a lower Rap1-GTP activity (18.85, arbitrary units, a.u.) than the wt retina (38.85, a.u.). In cultured retinas the activator S-220 (20.92 a.u.) augmented Rap1-GTPactivity compared to Ctrl (10.80 a.u.) while ESI-05 lowered the activity (7.00 a.u.). Bottom panel) Full SDS-PAGE and western blots from which data in the upper panel was derived. The figure represents, 10-16 pulled retinas per condition and the outcome is represented with an arbitrary unit value per model. No statistic method was applied due to only one data point per condition.*
